# Reoperations After Cataract Surgery: Is the Incidence Predictable Through a Risk Factor Stratification System?

**DOI:** 10.7759/cureus.10693

**Published:** 2020-09-28

**Authors:** Eirini Oustoglou, Argyrios Tzamalis, Ioannis Mamais, Maria Dermenoudi, Konstantinos T Tsaousis, Nikolaos Ziakas, Ioannis Tsinopoulos

**Affiliations:** 1 2nd Department of Ophthalmology, Papageorgiou General Hospital/Aristotle University of Thessaloniki, Thessaloniki, GRC; 2 Department of Hygiene, Epidemiology and Medical Statistics, National and Kapodistrian University of Athens, Medical School, Athens, Greece, Athens, GRC; 3 Department of Health and Life Sciences/Epidemiology, Biostatistics and Methodology Research, European University of Cyprus, Nicosia, CYP; 4 Department of Ophthalmology, Health Center of Neapolis, Thessaloniki, GRC; 5 2nd Department of Ophthalmology, Papageorgiou General Hospital/Aristotle University of Thessaloniki, Thessaliniki, GRC

**Keywords:** cataract surgery, complications, reoperations, risk factors, stratification system

## Abstract

Introduction

The objective of the study was to quantify the number of procedures needed to achieve the best possible surgical outcome, depending on the number and type of risk factors identified.

Methods

Two independent observers reviewed the medical records of 1,502 patients who underwent phacoemulsification surgery, during a two-year period (January 1, 2014 to December 31, 2015). Preoperative risk factors were documented according to the stratification system used. Based on the total risk score, each case was allocated to one of four risk groups with 0, 1-2, 3-5, and >6 total risk factors, respectively. All qualitative and quantitative characteristics were gathered and included in a multivariate analysis.

Results

A total of 1,792 eyes were included. Αge over 88 years, low cooperation ability with the patient, and surgeries performed by residents tended to have more often complications, while white/intumescent cataract, iridodonesis/phacodonesis, α1 blockers intake, and male gender are risk factors positively associated with more than one surgery.

Conclusions

Risk factors tend to be prognostic for possible intraoperative complications. The number of procedures needed for the best possible surgical outcome seems to depend on these preoperative risk factors. A stratification method increases the level of awareness of the surgeon, and therefore may decrease the number of complications and even procedures while enhancing the "safe" practice and skills of residents.

## Introduction

Age-related cataract is a well-known and described major public health problem. As the leading cause of blindness worldwide it has a direct impact on the economic, social life, and public health in general [1,2]. Although cataract surgery has been proved to be an effective treatment, its cost and the lack of well-trained surgeons limit its availability in many parts of the world, contributing to its leading course of blindness, especially in developing countries.

Despite the severity of the issue, public health systems worldwide are trying to cut off expenses in every possible sector. In an effort to cut off expenses of cataract surgery without compromising patients’ safety, numerous groups of researchers have attempted to establish whether certain factors, evident at preoperative assessment, might be useful predictors of an intraoperative complication [3]. Being able to predict and avoid any possible complications is a good way to reduce the costs without any reduction in healthcare quality. In our study, we used a previously validated scoring system for assessing the risk of intraoperative complications in patients undergoing cataract surgery, to quantify the number and type of procedures needed for the best surgical outcome [4-7]. There are no national data available to compare to our own yet. The main purpose was to quantify the number of procedures needed to achieve the best possible surgical outcome, based on the number and type of the risk factors identified preoperatively, and highlight the influence of the risk factors to the difficulties faced intraoperatively.

## Materials and methods

We conducted a retrospective cohort study by reviewing the medical records of all patients undergoing phacoemulsification surgery during a two-year period (January 1, 2014 to December 31, 2015). The ethical permission of the study was approved by the Scientific Committee of Papageorgiou General Hospital, Personal Data Protection Authority, the Third Health Region of Macedonia and adhered to the declaration of Helsinki tenets. We searched out for post-surgery complications indicative of the conduction of additional surgery, such as early sunset syndrome, retained lens fragments, and ocular hypertension as well as retinal detachment, for a period of three months post-surgery. 

Inclusion criteria involved all cataract patients operated by means of phacoemulsification in the time of interest. Exclusion criteria were cataracts beyond age-related reasons such as congenital [8], traumatic [9], drug induced [10-12], and due to inflammatory causes [13]. Four cases of post-cataract surgery endophthalmitis were also excluded as irrelevant to the aim of the study. Their medical files were examined, and no intraoperative complication was found or any preoperative risk. The range of complications recorded were incomplete capsulorhexis, posterior capsule rupture, zonulae dehiscence, retained nucleus, and anterior capsule tear with or without vitreous loss. All additional surgeries were recorded, and causes were analyzed accordingly. 

The additional procedures followed were decided either at the end of the first surgery or postoperatively according to our departmental guidelines. The type of additional procedures needed were relocation of dislocated intraocular lenses (IOLs), retained lens fragments removal with or without IOL implantation, pars plana vitrectomy for retinal detachment or cryotherapy in case of retinal tear, anterior chamber IOL implantation, relocation of postoperative iris prolapses, and in case of poor cooperation of the patient, surgery in general anesthesia. 

At the preoperative check, all risk factors were documented on a specific form along with patient’s medical history and measurements with the IOL Master 500 (Zeiss, Oberkochen, Germany). Each intraoperative difficulty and complications were recorded on the electronic OR protocol, shortly after the end of surgery along with measurements from the CENTURION® Vision System (Alcon, Fort Worth, TX, USA) such as total phaco time and cumulative dissipated energy.

Based on the scoring system [7] used, previous vitrectomy [14,15], small pupil (<3 mm) [16,17], shallow anterior chamber (depth <2.5 mm) [18], age >88 years [19-21], high ametropia (>6 D of myopia or hyperopia) [22], pseudoexfoliation (PEX) [23,24], white/intumescent cataract [25,26], posterior polar cataract [15], phacodonesis/iridodonesis [15], miscellaneous risks assessed by the surgeon at the pre-operative check (poor cooperation or position of eye/patient [15,27], medication such as a1 blockers [28,29], and corneal scarring [15] were described and documented preop. Each one of them was given one score point except for white/intumescent cataract, PEX, and phacodonesis/iridodonesis, which were given three points. According to the total points of risk accumulated using this system, the patients were preoperatively allocated to one of four risk groups: group Α (no risk factors present), group B (risk factor score = 1-2), group C (risk factor score = 3-5), and group D (risk factor score ≥6), and then cases were assigned accordingly to residents and consultants of the hospital based on their experience in cataract surgery. Residents operated on cases with no risk factors, except for medically substantiated diabetes and glaucoma. Residents had experience of up to 100 surgeries and the consultants more than 500. 

The chi-square test and Fisher's exact test were used to compare frequency between the groups, and Mann Whitney U-test was used to compare group means. All tests were two-sided, and the significance levels were set at 0.05. Logistic regression analysis was used to evaluate the odds ratios (ORs) and 95% confidence intervals. Analyses were performed using the SPSS statistical package (SPSS Inc., Chicago, IL, USA).

## Results

Overall, 1,502 patients (1,792 eyes) were enrolled in this study. A three-month follow-up was recorded for every case until March 31, 2016 to seek any postop complications and additional surgeries. There were 944 women (52.7%) with a mean age of 73.29 (SD 9.03) and 848 men (47.3%) with a mean age of 72.17 (SD 9.44) years (p=0.011). Men had a significantly higher (p<0.001) percentage of complications (13.7%) compared to women (7.8%). Within the complicated cases, men had to do at least one additional surgery in 2.4% (p=0.002) compared to 0.6% of complicated cases on women. The mean patient age was 72.76 years (range 32-94, SD 9.24), and 49.2% of phacoemulsification surgeries were conducted to left eyes. Residents performed 12.3% of the total number of surgeries, and the rest were handled by consultants and fellows of the department. 

Given the stratification system used preoperatively, 52.6% of the cases belonged to group A (zero total scores), 24% to group B, 20% to group C, and 3.2% to group D. The maximum score of risk factors found was 10 in 0.1% of our cataract patients. 

**Figure 1:**
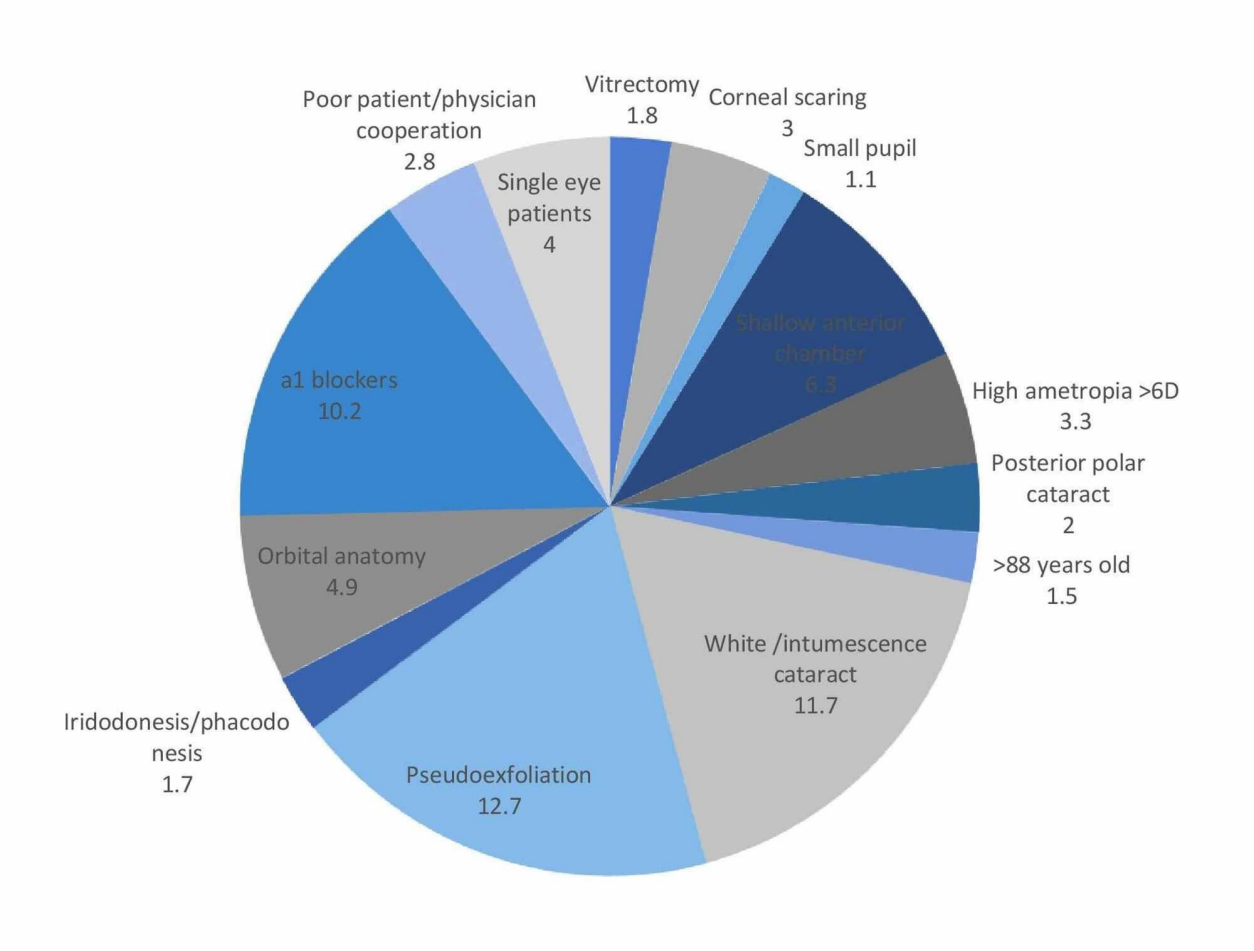
Risk factor rates

The most common risk factor found was PEX in 12.7% (227 eyes) of all cases, white/intumescent cataract in 11.7% (210 eyes), and a1 blocker intake in 10.2% (183 eyes). All the other risk factors did not exceed 10% of the cases (Figure 1).

**Figure 2:**
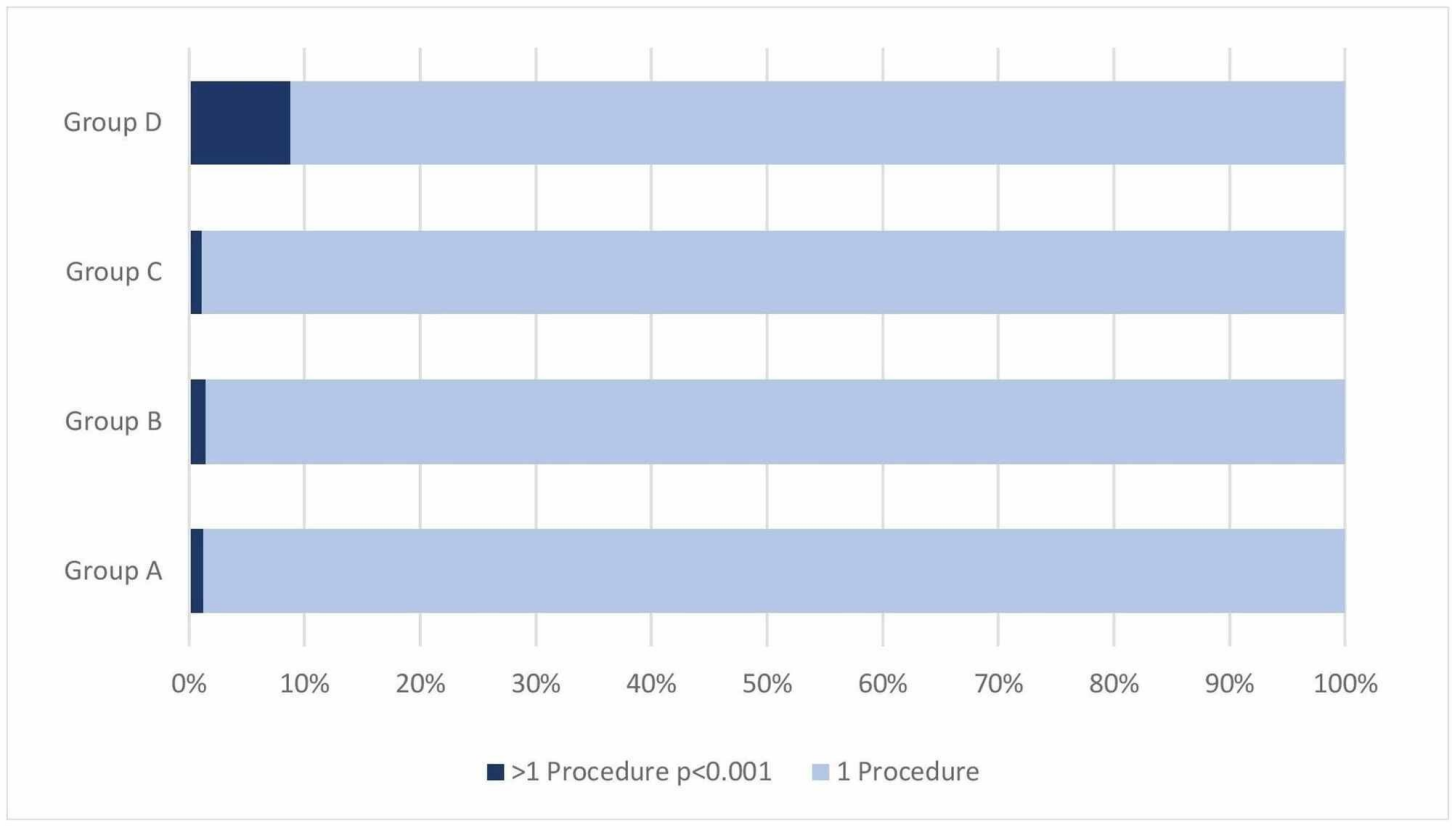
Relative frequency in >1 procedure by risk group

A positive correlation between the risk group and the complication rate was found (Pearson’s chi-squared test, p=0.003), starting with risk factor 0 group having 9.2% complications, group B having 11.6%, group C having 11%, and finally group D having 24.6% complications. Following the complication rate, a positive correlation in conducting an additional surgery was found to be significantly important between the risk groups (Pearson’s chi-squared test, p<0.001) (Figure 2). 

Every variable was tested independently for its statistical value regarding complications and additional procedures. Male gender (p<0.001), total risk factor score (p=0.003), age >88 years (p<0.001), white/intumescent cataract (p<0.001), iridodonesis/phacodonesis (p=0.002), a1 blockers (p=0.029), poor patient/physician cooperation (p=0.034), and resident-performed surgery (p<0.001) have exceeded the level of statistical significance as risk factors in complicated cases (Table 1).

**Table 1:** Risk factors and statistical significance in complicated cases

Risk Factors	Complication Rate	P Value
White/intumescent cataract	38 (20.0%)	<0.001
Iridodonesis/phacodonesis	7 (3.68%)	0.022
a1 blockers	28 (14.74%)	0.029
Total score ≥6	14 (7.37%)	0.003
Male gender	116 (61.05%)	<0.001
Residents	52 (27.37%)	<0.001
Age >88 years	9 (4.74%)	<0.001
Poor patient/physician cooperation	10 (5.26%)	0.034
Vitrectomy	5 (2.63%)	0.392
Corneal scars	6 (3.16%)	0.863
Poor pupil dilation <3 mm	3 (1.58%)	0.521
Shallow anterior chamber <2.5 mm	13 (6.84%)	0.721
Ametropia >6D	2 (1.05%)	0.063
Posterior polar cataract	4 (2.11%)	0.920
Pseudoexfoliation	23 (12.11%)	0.805
Orbital anatomy (deep-seated orbits)	13 (6.84%)	0.193
Single eye patients	4 (2.11%)	0.165

In a multivariable regression analysis, fewer factors such as male gender (OR 2.01), residents (OR 4.65), total score 1-2 (OR 1.02), total score 3-5 (OR 2.49), total score >6 (OR 8.33), PEX (OR 0.48), and age (OR 1.02) reached the level of statistical importance (Table 2). 

**Table 2:** Multivariable regression analysis/dependent variable complication existence

Risk Factors	Odds Ratio	P Value	95% CI	
Male gender	2.01	0.000	1.46	2.78
Residents	4.65	0.000	3.08	7.02
Total score 1-2	1.02	0.014	1.01	1.04
Total score 3-5	2.49	0.000	1.51	4.11
Total score 6	8.33	0.000	3.58	19.39
Age	1.02	0.014	1.01	1.04
Pseudoexfoliation	0.48	0.022	0.25	0.89

Regarding miscellaneous risks assessed by the surgeon at the preoperative check, deep sulcus (p=0.509), reduced cooperation ability (p=0.757), and single eye patients (p=0.297) did not reach levels of statistical significance except for a1 blockers (p=0.029). 

From the 190 complicated cases, 26 (13.7%) required additional procedures, which represents 1.5% (p<0.001) of all surgeries. In an attempt to correlate the cases with more than one surgery, with the risk factors preoperatively documented, variables were tested independently. According to the results, significant risk factors were white/intumescent cataract (p=0.002), iridodonesis/phacodonesis (p<0.001), a1 blockers (p=0.029), male gender (p=0.002), and total score >6 (p<0.001) (Table 3). 

**Table 3:** Risk factors and additional procedures

Risk Factors	Frequency	>1 Surgeries	P Value
White/intumescent cataract	210 (11.7%)	8 (3.8%)	0.002
Iridodonesis/phacodonesis	30 (1.7%)	3 (11.5%)	<0.001
a1 blockers	183 (10.2%)	6 (23.1%)	0.029
Total score ≥6	57 (3.2%)	5 (8.8%)	<0.001
Male gender	848 (47.3%)	20 (2.4%)	0.002
Vitrectomy	33 (1.8%)	1 (3.8%)	0.444
Corneal scars	53 (3%)	0 (0%)	0.370
Poor pupil dilation <3 mm	20 (1.1%)	0 (0%)	0.585
Shallow anterior chamber <2.5 mm	112 (6.3%)	2 (7.7%)	0.760
Ametropia >6D	60 (3.3%)	0 (0%)	0.339
Posterior polar cataract	36 (2%)	1 (3.8%)	0.501
Age >88 years	26 (1.5%)	1 (3.8%)	0.304
Pseudoexfoliation	227 (12.7)	4 (15.4%)	0.675
Orbital anatomy (deep-seated orbits)	88 (4.9%)	2 (7.7%)	0.509
Poor patient/physician cooperation	51 (2.8%)	1 (3.8%)	0.757
Single eye patients	71 (4%)	0 (0%)	0.297
Residents	220 (12.3%)	3 (11.5%)	0.908

In the logistic regression analysis conducted, male gender, total score, white/intumescent cataract, iridodonesis/phacodonesis, and a1 blockers reached levels of statistical significance, with the number of surgeries as the dependent variable. We also proceeded to a multivariable logistic regression analysis, using the stepwise method, where we found that the presence of white/intumescent cataract increased the risk of additional surgeries by 2.94 times (p=0.017), iridodonesis/phacodonesis by 5.35 times (p=0.013), and a1 antagonists by 2.71 times (p=0.037) (Table 4).

**Table 4:** Multivariable regression analysis/depended variable >1.1 surgeries

>1 Procedures	Odds Ratio	P Value	95% CI	
White/intumescent cataract	2.94	0.017	1.21	7.12
Iridodonesis/phacodonesis	5.35	0.013	1.43	20.06
α1 blockers	2.71	0.037	1.06	6.90

Finally, a chi-square test was used to determine whether the complication rates or the need for additional procedures differ between the vitreoretinal (VR) and the cataract surgeons. As a common practice in Greece, every VR surgeon performs also cataract surgeries in public hospitals and private practice. In our setting, four out of eight are VR surgeons and performed nearly 18% of total phacoemulsification surgeries. A hypothesis was made on how the difference in specialty interferes with the surgical technique in complicated cases to achieve the best possible surgical outcome. Within our data, the level of significance was not reached either in the complication rates (p=0.141) or the extra procedures (p=0.247) between the two groups of surgeons. 

## Discussion

The existence of a stratification method in preoperative assessment of cataract surgery is beyond doubt of outmost importance. It is a way to be prepared to avoid intraoperative complications or manage to handle them in the best way possible. Through low-risk cases, residents can evolve their skills through "safe" practice.

According to international data, white/dense mature cataracts might increase the level of difficulty particularly in the stage of continuous capsulorhexis, mean phacoemulsification time, and energy needed [25]. These factors can cause posterior capsule rupture with a negative effect on visual acuity [26] and, as far as our results support, may influence the risk of additional surgery almost three times.

Another risk factor suspected to interfere with the probability of an additional surgery, at least by five times according to our results, is iridodonesis/phacodonesis. International literature supports the high complication rate in these cases and points out the right preoperational assessment to be of great importance in the hands of experienced surgeons [15], as these cases are inappropriate for teaching purposes. 

Intraoperative floppy iris syndrome (IFIS) is a very common incident between patients receiving a1 blockers and usually requires modifications in surgical techniques and an experienced surgeon available in time of need [27]. Approximately 1.5% of patients receiving these drugs had complications that requested additional surgery. At the same time, finding a statistically significant relationship between the number of surgeries and male gender does not appear to be affected by the statistically significant effect of a1 antagonists in the total of 848 men, of whom 180 (21.22%) received these substances. The other three cases with IFIS and complicated cataracts having additional surgeries were women with urological issues or benzodiazepines intake which works as a reminder of the need for a well-structured sex-independent preoperative check [28]. In our study, the male gender had a statistically significant role in complications and additional surgeries which, at the moment, cannot be explained by any scientific documentation and contradicts some other reports regarding the number of complications [16]. 

Resident cases had no risk score, except for diabetes and glaucoma, and represented 12.3% of total surgeries. The complication rate was 23.6%, almost five times higher than in cases performed by fellows or experienced consultants. Despite the increased complication rate, there was no statistical significance between the number of surgeries and resident surgeons, probably because when a case was complicated by certain events listed above the surgery was finished by the attending experienced surgeon. Nevertheless, this increased rate should be considered and risk factors such as diabetes and glaucoma may need to be correlated with the complications and be integrated to the risk factor list. 

The multivariable regression analysis used found an increased risk of complications between groups. Total scores of 1-2 (group B) had no more than 2% increased risk of complications compared to the risk-free group (group A). Group C increased its risk of complications by almost 2.5 times and the last group of total scores above 6 (group D) had 8.33 times the risk of complications of the group of zero total scores. Fewer risk factors seem to link to a safer postoperating outcome. 

Approximately 52.6% of patients belonged to the risk factor-free group because of the structure of the National Care System which allows everyone to seek medical care in a tertiary hospital.

The age of the patient reaching the statistical significance level in terms of complications was associated with a 2% risk for every year passed, starting from the age of 32 years (which was the minimum age in our study). This is a well-described risk factor for its contribution to the complications of the phacoemulsification technique alone [16,19,21] or combined with other risk factors [20], agreeing to the study outcome, but not contributing to a significant level to the additional procedures needed. The evolution of technology and open access to information makes people in developed countries more aware and may contribute to the decrease in the mean age of conducting cataract surgery in the future.

The demarcation between VR and cataract surgeons did not come up with statistical significant results. Choosing a different day to optimize a surgical outcome seems to serve both groups in case of preoperative design and effectiveness of surgery.

An interesting observation in the results is the 0.48 risk of complications in cases with PEX. It appears as a protective factor with a 0.52 success rate. Studies all over the world recognize PEX as a risk factor to be in mind during surgery to avoid complications [23,24]. The geographic distribution of PEX is favoring surgeons to familiarize and master the techniques needed to prevent complications in the existence of such a factor. A modification to the stratification system used may include the reduction of the risk assessment at least for the regions of a wide distribution of the factor. 

The findings in the study are limited by the absence of stratification in the group of consultants. In this case, the complication rate is higher than those commonly described internationally. A stratification based on surgical experience should be conducted to avoid any misleading conclusions. The optimal postoperative outcome was defined as “lege artis” surgical operation and treatment of any intraoperative complication considering the safety of the patient. However, the optimal outcome may also be related to the individual experience. There was no correlation with visual acuity postoperatively due to the low rate of postoperative records of best-corrected visual acuity (56.98%). 

There were no other national data available, analyzing the risk of additional surgeries in correlation to risk factors assessed preoperatively, to compare to our own. 

## Conclusions

An attempt of a direct correlation between risk factors and the number of procedures was made, showing male gender, total risk score, white/intumescent cataract, iridodonesis/phacodonesis, and α1 blockers intake, influencing significantly the surgical result. In the same time, PEX should be always considered an important risk factor but may be scored according to its geographical prevalence.

Taking into account such risk factors allows proper preoperative preparation to limit intraoperative complications in every surgical step and more accurate patient information on postoperative risks. The surgeons acknowledge the “power” of each preoperative factor in the postoperative result and therefore increase their level of awareness intraoperatively while promoting the educational process in ophthalmological departments.
